# Public policies and health systems in Sahelian Africa: theoretical context and empirical specificity

**DOI:** 10.1186/1472-6963-15-S3-S3

**Published:** 2015-11-06

**Authors:** Jean-Pierre Olivier de Sardan, Valéry Ridde

**Affiliations:** 1 Laboratoire d'Etudes et de Recherche sur les Dynamiques Sociales et le Développement Local (LASDEL), BP 12901, Niamey, Niger; 2 École des Hautes Etudes en Sciences Sociales (EHESS), Paris, France; 3 University of Montreal School of Public Health, 7101 Avenue du Parc, 3rd Floor, Montréal QC H3N 1X9, Canada; 4 Institute of Public Health Research of University of Montreal (IRSPUM), Montreal, Canada

## Abstract

This research on user fee removal in three African countries is located at the interface of public policy analysis and health systems research. Public policy analysis has gradually become a vast and multifaceted area of research consisting of a number of perspectives. But the context of public policies in Sahelian Africa has some specific characteristics. They are largely shaped by international institutions and development agencies, on the basis of very common 'one-size-fits-all' models; the practical norms that govern the actual behaviour of employees are far removed from official norms; public goods and services are co-delivered by a string of different actors and institutions, with little coordination between them; the State is widely regarded by the majority of citizens as untrustworthy. In such a context, setting up and implementing health user fee exemptions in Burkina Faso, Mali and Niger was beset by major problems, lack of coherence and bottlenecks that affect public policy-making and implementation in these countries.

Health systems research for its part started to gain momentum less than twenty years ago and is becoming a discipline in its own right. But French-speaking African countries scarcely feature in it, and social sciences are not yet fully integrated. This special issue wants to fill the gap. In the Sahel, the bad health indicators reflect a combination of converging factors: lack of health centres, skilled staff, and resources; bad quality of care delivery, corruption, mismanagement; absence of any social security or meaningful commitment to the worst-off; growing competition from drug peddlers on one side, from private clinics on the other. Most reforms of the health system have various 'blind spots'. They do not take in account the daily reality of its functioning, its actual governance, the implicit rationales of the actors involved, and the quality of healthcare provision. In order to document the numerous neglected problems of the health system, a combination of quantitative and qualitative methods is needed to produce evidence.

## Introduction

The empirical basis of our research is provided by Sahelian Africa, with the focus on a specific type of health policy (the recent wave of user fee exemptions in Africa). The main findings have already been published in French [1] and in English [2]. They are summarised in the present Introduction (Olivier de Sardan & Ridde, this issue). The literature on the switch from cost recovery to user fee exemptions is discussed in Ridde (this issue).

However our interpretative framework is much broader. This contribution presents the theoretical context of this special issue, which is located at the interface of two major research areas that have developed on an international scale over the last 30 years: public policy analysis and the study of health systems. They are now often combined under the same heading: Health Policy and System Research (HPSR).

While health systems have received increasing attention in recent years, health policies are still very much neglected, particularly in Africa. As Gilson & Raphaely put it: "Health policy analysis in LMICs clearly remains in its infancy" [3]. Our research is an attempt to fill this gap. But this means taking not only health policy studies, but also, more generally, public policies studies into account.

Thus, in this paper we provide a summary of the theoretical setting concerning, first, public policy literature and, second, health system literature, in order to answer the same two questions for each area: (a) What kind of research is being undertaken at international level and where does our own research stand in relation to it? (b) To what extent is the situation in Africa peculiar to that continent and what are the implications for our research?

One thing should be made clear from the outset: any feature of public policy-making and health systems in Sahelian Africa (and, in our own experience, in West Africa generally) could also be found in some way or another in Europe and North America, but in different proportions and with different styles. Bureaucracy in the health system is a case in point. Nurses in African health centres complain (mostly with good reason) about the huge number of reports and other documents they have to complete on a daily basis and at the end of every month. But in her analysis of bureaucracy in a French hospital, Béatrice Hibou describes how a Parisian nurse also rails against these very ills, having to complete mounds of forms - which are often irrelevant to her work and useful only to managers - before she can even start her clinical work [4]. There is a need for comparative analyses of African and European bureaucracy, a fact which justifies the application of the same research perspectives to both the southern and northern hemispheres [5]. However, it does not mean that contexts are the same - on the contrary.

For example, there is evidence that the behaviour of health professionals diverges from official norms everywhere, but the extent, frequency and nature of these divergences vary considerably, depending on the context. At the present time, they are often more significant in Niger or Mali than in Sweden or Germany. In principle, the aim of every public policy is coherence and effectiveness. In no country in the world is this aim achieved completely, but incoherence, implementation gap and policy failure are often greater in Sahelian Africa than in Europe, as the whole Africanist literature in political science shows, whatever the interpretations - often contradictory - provided for this phenomena. Every health system is composed of interdependent, regulated elements. Each one of them has bottlenecks and contradictions, but these are often more common and more severe in Sahelian Africa than in North America, even if the health systems of Canada and the United States are far from perfect.

### Public policies

#### Public policy research

Public policy analysis has gradually become a vast and multifaceted area of research in its own right, consisting of a number of perspectives and even paradigms, on which we have drawn to varying degrees. Eight of these are presented below (there are other dimensions to public policy research, relating, in particular, to vested interests or the institutions involved, that we cannot take in account here). These perspectives are in no way incompatible, they frequently overlap, and are often mixed (as we did).

##### 1. Sequences

The best known is probably *sequential analysis *(deLeon's *stages heuristic*) with its discrete phases, which have become a standard feature of the 'public policy process': agenda-setting, policy formulation, legitimation, implementation, and evaluation [6]. Having been criticized for its linear approach [7], sequential analysis has become more sophisticated and dialectical, taking account of the 'garbage can' model [8] and of interactions between 'stages' and between 'streams' [9] and adopting a standpoint more focused on process and dynamics [10,11]. It continues to underpin numerous studies [12]. Kingdon's policy streams framework is often used in the area of health [13]. For our part, we made a basic, two-part distinction: (a) the *shaping *of policies, in other words agenda-setting, decision-making, legitimation and formulation (one of the paradoxes of free healthcare policies, especially in the case of Mali and Niger, is that legitimation has preceded formulation, in contrast to the usual order of these stages, which means that a wider perspective has to be adopted); and (b) their *implementation*. As for the analysis of outcomes, this can be included under implementation or else dealt with separately.

##### 2. Agenda-setting and decision-making

The emergence of a problem as a 'public problem' and the ways in which it is incorporated into a political agenda have generated a great deal of literature [14]. We have documented how the free healthcare paradigm, which, in terms of public health, had assumed increasing prominence on the world stage after being ignored for a long time [15], has suddenly been adopted by heads of African states [2] (Ridde, this issue).

##### 3. Implementation

The 'implementation' of policies has long been the point of entry to a specific research current that is particularly relevant here. Since Pressman & Wildavsky's famous book [16], and to some extent before it [17], '*implementation studies*' have focused in different ways on the '*implementation gap*', in other words on the disparity between public policies as decided, developed and organized (their aims and architecture) and what actually happens on the ground (the facts surrounding their implementation, and how they are appropriated/misappropriated/transformed/dismantled in practice). This approach has its origins in the political and administrative sciences, and is still largely concerned with the northern hemisphere [3,17]. Following an initial phase dominated by 'top-down' perspectives, it became more sensitive to the entanglement of many logics and various stakeholders, including users and frontline bureaucrats, feedback, and informal process of negotiation and bargaining [18-21]. However, in our view, it neglected a methodological tradition of intensive fieldwork, especially in Africa, for the in-depth investigation of such issues. The new anthropology of development filled this gap over the course of the 1980s, when it adopted the implementation approach in relation with Africa and aid policies [21,22], giving it a more detailed and robust empirical content by analysing the interactions between 'developees' and 'developers', and the 'drifts' and unexpected outcomes of development projects. It did so by exploiting the methods of ethnography (qualitative fieldwork), which also play a prominent part in our own study. Moreover, both the approach and the method have since been used by the anthropology of the State and of public action in Africa [5] which is a contemporary widening of the anthropology of development (including health services provided by the State), and a domain in which a number of our own researchers work.

It will be seen below just how important this *implementation gap *is in relation to free healthcare policies in the three West African countries investigated. This has been the main thrust of our research. Various studies have analysed these considerable divergences between official policies and actual practice in other health sectors [23-25]. However, it should not be forgotten that the situation is not confined to health. A similar gulf can be found in virtually all public policies in these countries, regardless of the sector. All recent research undertaken within an ethnographical framework on justice [26,27], education [28,29] and decentralization [30-32] in Africa bears witness to the fact.

##### 4. Frames of reference

Other public policy research has chosen to focus on 'frames of reference' (*référentiels *in French) for these policies, in other words on the discursive, ideological and representational mechanisms that underpin the design and agenda-setting stages of these policies, allowing them to be thought out and articulated, and either explicitly or implicitly legitimizing [33-35]. We have paid some attention to this issue ourselves regarding the emergence and formulation of recent user fee exemptions [2] (cf. also Ridde, this issue). A peculiarity of public policies in French-speaking West Africa is that their frames of reference are developed in the main by experts from the northern hemisphere within a 'developmental perspective' [22]. This was true of cost recovery (Bamako Initiative) in the 1980s, introduced by UNICEF and WHO, and, from the first years of the 21st century, partly true for fee exemptions and for universal health coverage, which were debated by international NGOs (and subsequently by cooperation agencies, especially the DFID - the department responsible for British cooperation) after they had gradually developed campaigns in these areas, which have gathered momentum over the years and are now supported by all international institutions. For its own internal reasons, South Africa, on the other hand, introduced free healthcare as early as 1994. Moreover, the fact that the framework of reference is external does not preclude decisions being taken for internal policy reasons, as it is apparent in the case of user fee exemptions.

##### 5. Instruments

The analysis of public policies 'through their instruments' has also been developed recently [36,37], from a number of angles. The term 'instrument' can have different meanings. Howlett [38], for example, uses it in a very broad sense. The restricted sense we adopt here relates to the technical support that has been developed for a given policy to run smoothly: 'material' or bureaucratic tools [39], or formal procedures (such as the 'logical framework') [40]. We have paid particular attention to the 'paperwork' of free healthcare (record cards, ledgers, notebooks), as well as the complicated paths taken by 'free care' invoices between services and ministries.

##### 6. Actors

Public policies can also be approached through their actors: decision-makers, experts, bureaucrats, technicians, field agents, paramedics, brokers, users and community representatives. Lipsky's book on 'street-level bureaucrats' [41] is a seminal contribution to this approach. An entire literature in sociology or the anthropology of professions could be referred to, in which the medical professions, in particular, feature prominently, the main point of reference being Freidson [42]. Some of them have been studied in an African context [43,44]. The approach via actors could also be compared to the approach via 'stakeholders' (*stakeholder analysis*) or via 'strategic groups' [45], which then link up with the different interests involved in public policy-making. Our own research was, of course, conducted among all health professionals associated with exemption, first and foremost those engaged in clinical work: nurses (who are usually the front-line carers), midwives, doctors and surgeons; but we have also focussed our attention on support staff (traditional birth attendants, ward assistants, carers, paramedics and managers), as well as supervisory staff (district management teams, regional boards) and civil servants in the health ministries. However, we did not favour a particular entry point nor did we target a specific profession. Naturally, we also interviewed many actors outside the health profession, such as the members of management committees and users.

##### 7. Inequalities

Various projects on public policy are concerned with the relationship between the policies and the inequalities that characterize the societies in which they are implemented, whether from a neo-Marxist perspective [46] or with a special emphasis on exclusion, inequities and vulnerability [47]. How do public policies reproduce or exacerbate social divisions, or, on the contrary, reduce or cushion their effects? The policy of cost recovery in health facilities has been criticized for developing inequalities and excluding the most vulnerable [15,48]. Fee exemptions, whether in relation to specific illnesses whose costs cannot be borne by the poorest (tuberculosis, HIV-AIDS) or in favour of categories regarded as especially vulnerable (pregnant women and children) are, of course, at the centre of such questions. They immediately become located within 'social' strategies focused on access to healthcare by the poor, in which the challenges of the political economics of targeting come to the fore [49]. Part of our research has consisted in assessing whether visits to health clinics by vulnerable groups (the bottom quintile in the classification of population based on income) have been made any easier by free healthcare policies [50].

##### 8. Deliberative process

Finally, an analysis of the deliberative processes relating to public policies is another approach we have used. It includes public debates, discussions by experts, conferences, general assemblies, citizens' juries, media coverage, polls, etc. (in the area of health, cf. Boyco et al.) [51]. We monitored and analysed the national conferences on free healthcare organized in Mali and Niger [52] and, in tandem with this, read through and analysed all the newspaper articles on the subject in both countries [53]. In this issue (Olivier de Sardan), we describe some difficulties encountered in Mali and Niger with health officials concerning the diffusion of our results.

#### The specific character of public policies in the Sahel

In terms of public policies, the general situation is much the same in Burkina Faso, Mali and Niger, not only in the health sector but in other areas too. The same shortfall in tax revenues makes them dependent on development aid (between 30 and 50 per cent of national budgets comes from foreign funding). Hence, the main public policies are shaped largely by international institutions and development agencies: as a result, they are very similar from country to country, the transfer of 'one-size-fits-all' and 'blue-print' policies becoming ever more common at the expense of their fitness for complex local realities [54,55]. The structural adjustment programmes of the 1980s seriously weakened public services and administrations; corruption has become widespread and public employees are often demotivated. The practical norms and professional cultures [56] that govern the actual behaviour of employees are far removed from official norms. On the ground, public goods and services are co-delivered by a string of different actors and institutions, with little coordination between them, the co-delivery itself pointing to distinct local forms of governance [57,58]. The State is widely regarded by the majority of citizens as untrustworthy, thus making populations sceptical about the ability of States to ensure the sustainability of any policy for which funding is not guaranteed by international donors. Thus, the data from Afrobarometer 2008/9 surveys conducted in 20 countries show that citizens of countries in Francophone West Africa (that is, Mali, Burkina Faso, Benin and Senegal) are the harshest judges of their governments with regard to their ability to provide health services [59]. The capacity of the national health system to reproduce the pilot projects of NGOs concerning free healthcare have likewise met with a great deal of scepticism [60,61] (cf. also Olivier de Sardan et al. in this issue).

The process of setting up and implementing user fee exemptions in Burkina Faso, Mali and Niger was beset by all the major problems that generally affect public policy-making in these countries. Based on our results, they can be summed up as follows:

- decisions taken suddenly and without proper preparation, partly under international pressure or persuasion but incorporating internal political preoccupations.

- no coordination between technical and financial partners, who are often ignorant of local conditions, sometimes absent from the scene in areas where their input is likely to be vital, and not accountable for the consequences of the measures they recommend.

- an architecture hastily developed and put in place by national technical experts from central government, often wrong-footed by the decisions of politicians;

- a failure to plan realistically, with no evaluation of a pilot stage (or without taking evaluations into account when they do exist), making it difficult to anticipate problems.

- late and insufficient information provided for the categories involved (health staff, health committees, local authorities and users).

- staff responsible for applying the new policies who are often opposed to them or in any case demotivated.

- a lack of coherence in applying them, and ad hoc measures that are ill-defined, inadequate and piecemeal and are added to existing arrangements without any attempt at harmonization.

- a lack of reliable monitoring, feedback and reporting of information about the problems encountered.

- the absence of any research infrastructure for evaluating and supporting public policies, and a failure to take account of research data in the implementation of policies.

- a tendency to recentralize decisions and funds when the decentralization process has only just begun.

If anyone needs convincing that health is far from being the only sector in which these conditions prevail, we might take two examples, one from a country featuring in our study, the other from a Central African country.

In Niger, decentralization came in 2004, amid pressure from two quarters, externally, from donors, and internally, from the Tuareg rebellion. However, the State has failed to honour practically any of the promises it had made to local government for years. For many years, it has not even passed on to them the taxes raised on their behalf. State officials (governors, prefects and technical services) have sought to use the municipalities to their own advantage, at the same time impeding the delegation of authority. The powers in health matters officially handed down to the municipalities have received neither financial nor technical support, and have therefore remained a dead letter.

The Democratic Republic of Congo is an extreme case, but an instructive one. An exemption policy has also been introduced there, but in the education sector, where the payment of school fees by parents had become the chief source of funding, throughout the school hierarchy. In contrast to the cost-recovery scheme for health, which was put in place in the 1980s and under which user fees are retained by the health centres (and used to buy medicines, pay the wages of a carer and a manager, and help with the running costs), only a small proportion of school fees are used at school level in the Congo, the rest being distributed along an ascending pathway that ends at the ministry itself. Under pressure from donors, the government suddenly decided to abolish these school fees without first preparing the ground, setting up the budgeting requirements or informing the teachers and parents. Accordingly, the corresponding practices did not disappear [62].

Clearly, this is not to claim that public policies are identical throughout Africa, regardless of sectors and countries. Instead, they share a 'family resemblance', in other words certain common or similar structural features, which are typical of an identical 'bureaucratic style of governance' and which no doubt relate to least two important political and institutional variables common to almost all African countries: (a) a colonial past, which was also the construction stage of a modern state apparatus of a very particular kind, an apparatus that remained in place after independence; (b) the key role later played by development aid and the rentier setting that it generates [63,64].

However, beyond this 'family resemblance', public policies take on a different complexion, depending on the domains and the national contexts with which they are connected. Moreover, as we have noted, a number of significant differences exist between the exemption policies pursued in Burkina Faso, Mali and Niger, respectively.

### Health systems

#### Health systems research

Although speeches on global health increasingly refer to health systems [65], research into health systems was relatively neglected by public health researchers for a long time, especially in French-speaking West Africa, where "The potential usefulness of research explicitly focused on health systems is under-rated" [66]. In 2000, a survey of the biggest database in the area of health (Medline) showed that only 0.7% of articles were related to research on health systems, with less than 5% of these dealing with countries in the Southern hemisphere. For a long time, the main preoccupation was with epidemiological studies on the description of diseases, their distribution in the population and how well their etiology was understood. In Africa, this corresponded with an expansion of vertical programmes for fighting diseases. Until very recently, therefore, an exclusive reliance on statistics and a lack of awareness of qualitative methods were the norm. Whenever health systems were studied, they were not analysed from a holistic perspective; instead, studies tended to concentrate on a specific aspect, such as care delivery or funding. Even in the area of funding, for example, studies undertaken in Africa were fragmented, with some homing in on revenue collection (direct payment or the willingness/ability to pay) and others on the procurement mechanisms of services taken in isolation. Research was focused more on health services than on health systems proper, viewed in their entirety. Anthropologists were called on to study only the so-called 'cultural' aspects of health, and economists to study only the cost-effectiveness of interventions.

Health systems research started to gain momentum in the early years of the twenty-first century. WHO's annual report for the year 2000 is one of the cornerstones of this shift in focus (followed by the 2008 report on primary healthcare). This report by WHO suggested ranking the world's health systems by performance, placing, for example, the countries covered by our research at the bottom of the table and France at the top. The use of quantitative indicators, which remains the basis for this type of comparative approach [67], and results in league tables, is often challenged [68]. However, WHO also declared that the aims of a health system are to meet a population's expectations (the concept of responsiveness) and to organize a fair financial contribution (hence taking into account people's ability to pay) in order to improve the health of the population, although insufficient emphasis was placed on social inequality in health matters in Africa and elsewhere [69]. Finally, WHO proposed an analytical framework for the strengthening of health systems, built around six essential functions (or 'building blocks'), which were initially four (and sometimes become eight): 1) health service provision; 2) health personnel; 3) health information; 4) medicines and vaccines; 5) the funding of the health system; and 6) governance and leadership [70]. In some countries, this framework has even become a dogma, which does not favour innovative, transverse or systemic analyses [71].

Various reforms of health systems also rely on this framework. But there is still too little research on the changes that these multiple and endless reforms engender in relation to the overall performance of health systems, and one might be forgiven for thinking that there is a distinct tendency for them to be guided more by ideology than by scientific evidence [4,72].

Today, however, health policy and systems research (HSPR) is becoming a discipline in its own right and is increasingly tapping into other disciplines (the administrative and management sciences, political sciences, anthropology, etc.). The first academic organisation dedicated to health systems research (Health Systems Global) was established in 2012, in the wake of two international conferences organized in 2010 (Montreux) and 2012 (Beijing), at which our research programme was represented. This trend is, of course, part of the bigger picture of the work done by the Alliance for Health Policy and Systems Research over the years.

However, two problems with HPSR, which our research attempted to tackle, should be mentioned here:

- first, there is still a "*tendency to under-value contributions to HPSR from social sciences*" [73]

- second, for a number of reasons, French-speaking African countries scarcely feature in it at all: few researchers, the majority of whom are not fluent in English; competition from consultation; and domination by a French research tradition in public health that is very medicine-orientated and epidemiological and not at all conducive to the emergence of interdisciplinary issues relating to policy and health systems (for example, at the Beijing (2012) and the Cape town (2014) world conferences on HPRS there were virtually no researchers from French institutions).

As a way of structuring this field, but also of increasing its visibility, field-specific journals have appeared in recent years (e.g. *BMC Health Services Research)*, as well as a variety of overview articles [73,74] and a reader presenting a selection of texts on health systems and offering a definition of the field [75]: "Health policy and systems research is defined as a field that seeks to understand and improve how societies organize themselves in achieving collective health goals and how different actors interact in the policy and implementation processes deployed to contribute to policy outcomes. By nature, it is interdisciplinary, a blend of economics, sociology, anthropology, political science, public health and epidemiology that together draw a comprehensive picture of how health systems respond and adapt to health policies, and how health policies can shape - and be shaped by - health systems and the broader determinants of health." [75]. It is interesting to note that this definition is very broad and links together - fortunately - health policy research (see above) and health systems research. The majority of these studies attempt to show that researchers need to move beyond traditional paradigm boundaries and marshal other theoretical concepts and approaches (for example *critical realism *[76] or *realist constructivism*' [77]) to gain a better understanding of complexity of health systems and the contexts in which they are implemented, and to exploit the complementarity of quantitative and qualitative methods from a mixed methods perspective. This is what we have tried to do in this research programme (see Ridde & Olivier de Sardan in this issue).

In a recent publication, WHO gives every indication of wanting to play an active part in driving forward health systems research [78]. It also appears, more generally, that actors in this field are particularly concerned about the use of outcomes and about how the gap between researchers and those responsible for health systems reform can be narrowed [79]. Our own work in French-speaking Africa belongs to this perspective [1,80].

#### The peculiarities of health systems in the Sahel

Although there has been a steady fall in mortality rates over the years in the three countries under consideration, especially as far as children are concerned, quantitative indicators for health remain very unsatisfactory and will not enable the Millennium Development Goals (MDG) to be attained by 2015 (cf. Ridde, this issue). This situation reflects a combination of four sets of converging factors.

1. Health centres are still far too few in number (problem of geographical accessibility) and remain poorly attended by patients; there are not enough skilled staff in rural areas, especially outlying areas; and the health system suffers from a chronic shortage of financial and material resources: the health budget is small and falls well short of international commitments (Abuja Declaration), the health centres are under-equipped, and small items of equipment, medicines and inputs are often in short supply.

2. The quality of care provided leaves much to be desired: contempt for the anonymous user, the extortion of money from patients, a lack of professional conscience, absenteeism, mismanagement of human resources, numerous shortcomings in managing inputs and stocks. These problems are regularly reported by users [23] and the press, but NGOs, international institutions and local politicians remain obstinately silent on the matter in the local arena.

3. In the absence of any social security or meaningful commitment to the worst-off (the official mechanisms for helping the destitute do not work very well, if they work at all), the low standard of living of the vast majority of the population soon transforms any spending on health (such as obstetric care) into 'catastrophic expenditure' (putting the economic viability of households at risk).

4. In all three countries, the public health system currently faces growing competition from two very distinctive types of modern private healthcare channels (apart from 'traditional' or, more often, 'neo-traditional' healthcare, either in the form of self-treatment or of the services of a variety of specialist 'healers', who are not necessarily any cheaper). For most people, both in the countryside and in towns, it is the 'informal pharmacies' ('*pharmacies par terre*'), in other words, informal vendors (peddlers or market stalls), who supply consumers with the majority of modern medicines. These are usually sold individually, without any kind of quality control and often smuggled into the country [81,82]. In urban areas and among the better-off, private clinics attract the more affluent clients.

In response to this situation, reforms of the health systems have become a regular phenomenon in Africa over the last 30 years. These have consisted of the promotion of primary healthcare, cost recovery, community participation (a resurgence of which is in evidence today), the establishment of districts and the health pyramid, hospital reform, the creation of mutual insurance companies, user fee exemptions, and, more recently, performance-based financing. To these should be added the innumerable and unending sector-based and vertical mini-reforms that constantly modify the organization of work: for example, there has been an explosion of healthcare programmes in the last 15 years in the area of mother-and-child health alone: emergency obstetric and neonatal care, essential newborn care, active management of the third stage of labour, refocused antenatal consultations, prevention of mother-to-child transmission of HIV-AIDS, clinic- and community-based integrated management of childhood illness, key family practices programme, rapid diagnostic tests for malaria, etc.

All of these mini-reforms are, in reality, mini-public-policies, on various scales. They correspond to what Hardee et al. [83] have called "operational policies", which are referred to as "programs" by other authors [84,85]. Indeed, every new public policy is presented as a reform of the policies in place. All of them are aimed at improving the current health system, at making it more effective and more efficient, and at providing a better service. Most of them are designed by experts from the North in the form of standard procedures to be implemented in many African countries. And yet, ironically, most of them do not start from a thorough diagnosis of the health system as it really is and actually works: the daily interactions between health workers and the population and the routine functioning of health services, which is often far removed from official norms and organizational charts.

In other words, most reforms and mini-reforms are based on the fiction that the health system in place in Burkina Faso, Mali and Niger is the official one and that health workers comply routinely to professional rules. The same thing happened in the case of fee exemptions.

Sadly, however, the reality is completely different. Users all complain about the way they are treated by health staff, about the corruption that is rife in health centres and about the fact that the care they receive is rushed and of poor quality. Low-level staff, who do the bulk of the work, bemoan the lack of equipment and shortages of supplies, the appropriation of bonuses and other benefits by those above them in the hierarchy, absenteeism among doctors, and even wheeling and dealing by some of them. Doctors fail to discipline staff who are found to be at fault, they fail to get midwives posted to outlying areas or to carry out checks on the quality of care, and are even more remiss in monitoring the application of the innumerable micro-reforms. Each of these micro-reforms is usually preceded by a short period of 'training': the health staff who attend are supposed to practise what they have learnt and disseminate it to their work colleagues. This rarely happens in reality.

Hence, the epidemiological studies and public health analyses undertaken in African countries have various 'blind spots'. In other words, apart from a few exceptions, a number of serious 'problems' in the day-to-day workings of health systems are hardly debated or 'brought out into the open', either because they are not picked up by the usual investigation protocols or because they have become firmly entrenched routines, or even because they challenge vested interests. And yet, many of the failures that bedevil health programmes are attributable precisely to these factors, which generally relate to the 'real practices' of health staff (which often diverge from official norms), the ways in which care is actually organized (with all their contradictions, inadequacies and inconsistencies), how health policies (often inconsistent themselves) are implemented on the ground (with significant discrepancies between intention and execution, and the use of extensive scope for manoeuvre by frontline workers).

## Conclusion

New field research needs to be done on these 'neglected problems', which concern the actual governance of health systems, the implicit rationales of the actors involved, and the quality (real or perceived) of healthcare provision. Posting and transfer (which are in reality very far removed from official regulations and optimal use of workforce) is an example of such "challenges that are all but ignored in the health literature" [86]. LASDEL (http://www.lasdel.net) is currently engaged in a research programme on 'Neglected problems of Niger health systems', which deals with six topics: supervision, midwives, medical records, induced abortions, what happens after the departure of medical NGOs, and the role of the municipalities in health matters. All these topics only appear in official documents in the form of slogans or instructions, and very rarely as complex issues needing to be documented. 'Evidence-based medicine', which is so fashionable nowadays, including in the area of public policies on health ('evidence-based policy'), seems only to regard what comes out of epidemiological investigations as 'evidence'. However, only finely tuned qualitative investigations are able to capture these neglected problems: these are a necessary complement to the work of a quantitative nature if the intention is to research into health systems in terms of their daily operations on the ground (cf. Ridde & Olivier de Sardan in this issue). It is precisely for this reason that we combined both types of investigation.

The first step towards indispensable reforms is to highlight the neglected problems faced by health systems and to document them. The fact is that, unless these problems are diagnosed - and most of the time they are not even mentioned publicly in national and international decision-making circles or in the field of epidemiology and public health - the implementation of health policies will continue to miss their intended targets by a wide margin, with a multitude of unintended and undesirable consequences. In the context of user fee exemptions the examples of these consequences are numerous [1] (see also the following articles by Touré and by Diarra/Ousseni in this issue).

## List of abbreviations

DFID : United Kingdom Department for International Development

HPSR : Health Policy and Systems Research

HIV-AIDS : Human immunodeficiency virus infection and acquired immune deficiency syndrome

LASDEL : Laboratoire d'Etudes et de Recherche sur les Dynamiques Sociales et le Développement Local

LMIC : Low and Middle Income Countries

MDG : Millenium Development Goals

NGO : Non Governmental Organisation

UNICEF: United Nations Children's Fund

WHO: World Health Organization

## Competing interests

None

## Authors' contributions

JPODS and VR conceived the idea, wrote the draft and final version of the manuscript. All authors read and approved the final manuscript.

## Declarations

This article has been modified from the chapters **Les spécificités des politiques publiques et des systèmes de santé en Afrique sahélienne** by J.-P. Olivier de Sardan and V. Ridde [87], and **La quantité sans la qualité ? Mises en forme et mises en oeuvre des politiques d'exemptions de paiements au Sahel** by J.-P. Olivier de Sardan [88] in the book *Une politique publique de santé et ses contradictions. La gratuité des soins au Burkina Faso, au Mali et au Niger*, J.-P. Olivier de Sardan and V. Ridde (eds), 2014, Karthala, Paris, with the permission of the publisher. The publication of this supplement was funded by a grant from International Development Research Centre (IDRC), Ottawa, Canada.

This article has been published as part of *BMC Health Services *Research Volume 15 Supplement 3, 2015: User Fee Exemption Policies. The full contents of the supplement are available online at http://www.biomedcentral.com/bmchealthservres/supplements/15/S3.
